# Dental Amalgam and Oral Biological Responses: A Narrative Review of Current Evidence

**DOI:** 10.3390/dj14030188

**Published:** 2026-03-23

**Authors:** Roxana-Cristina Mehedinti, Catalin-Bogdan Satala, Kamel Earar, Madalina Nicoleta Matei, Gabriel Valeriu Popa, Ada Stefanescu, Antoanela Magdalena Covaci, Roxana Adina Barascu Petrescu, Cristian Petcu, Dana Tutunaru

**Affiliations:** 1 Faculty of Medicine and Pharmacy, Medical and Pharmaceutical Research Center, “Dunarea de Jos” University of Galati, 800008 Galati, Romania; r_mehedinti@yahoo.com (R.-C.M.); kamel.earar@ugal.ro (K.E.); madalina.matei@ugal.ro (M.N.M.); gabriel.popa@ugal.ro (G.V.P.); ada.stefanescu@ugal.ro (A.S.); antoanela.covaci@ugal.ro (A.M.C.); dana.tutunaru@ugal.ro (D.T.); 2 The School for Doctoral Studies in Biomedical Sciences, “Dunărea de Jos” University of Galați, 800008 Galati, Romania; 3 Department of Pathology, Clinical County Emergency Hospital, 810325 Braila, Romania; 4 Clinical County Emergency Hospital, 010024 Craiova, Romania; roxanaadinapetrescu@yahoo.com (R.A.B.P.); cristipetcu80@yahoo.com (C.P.); 5 Clinical County Emergency Hospital “Sfantul Apostol Andrei”, 800578 Galati, Romania

**Keywords:** dental amalgam, mercury exposure, oral mucosa, salivary biomarkers, oxidative stress, inflammation, gingival pathology

## Abstract

Dental amalgam remains widely used in restorative dentistry due to its durability and cost-effectiveness, yet concerns persist regarding potential biological effects related to mercury release. This narrative review critically synthesizes current evidence on oral mucosal alterations and salivary biomarker changes reported in association with amalgam restorations. Experimental research supports biological plausibility for oxidative and inflammatory responses to mercury exposure; however, most human evidence derives from observational studies demonstrating heterogeneous associations rather than consistent causal relationships. Reported variations in salivary biomarkers, including interleukin-8 and ceruloplasmin, are generally modest and influenced by confounding factors such as periodontal status, smoking, and systemic inflammation. Histopathological findings adjacent to amalgam restorations include epithelial and inflammatory changes, though many are nonspecific and comparable to other chronic irritative conditions. Overall, current clinical and epidemiological data do not indicate uniform or clinically significant adverse effects in the general population attributable solely to dental amalgam. Regulatory phase-down initiatives primarily reflect environmental and precautionary policies. Available evidence supports a balanced and evidence-based interpretation of amalgam-related biological findings in contemporary dental practice.

## 1. Introduction

Dental amalgam has been used for more than a century as a restorative material in operative dentistry, representing one of the most durable, cost-effective, and technically accessible options for the treatment of posterior dental caries [1]. Traditionally composed of a mixture of elemental mercury combined with a powdered alloy of silver, tin, copper, and other trace metals, dental amalgam has long been valued for its mechanical strength, wear resistance, and longevity in the oral environment [2]. Owing to these characteristics, amalgam restorations continue to be present in a large proportion of adult patients worldwide, particularly in regions where public healthcare systems still rely on low-cost restorative solutions [3].

Despite its clinical advantages, the biocompatibility of dental amalgam has been a subject of persistent debate. The oral cavity represents a dynamic biochemical environment characterized by fluctuations in pH, temperature, mechanical loading, and microbial activity, all of which may influence the corrosion and degradation of metallic restorative materials. Under such conditions, dental amalgam is known to release small amounts of mercury in various forms, including metallic mercury vapor and ionic species. These products can be absorbed locally by the oral mucosa or systemically through inhalation and ingestion, raising concerns regarding their potential biological effects [4,5,6].

Mercury is a well-recognized toxic element, particularly in its organic forms and at high levels of exposure [7,8]. Its capacity to induce oxidative stress, interfere with enzymatic activity, and modulate immune responses has been extensively documented in experimental and toxicological research [9,10,11]. However, the biological implications of chronic low-level exposure specifically derived from dental amalgam restorations remain a subject of ongoing scientific debate. While mechanistic studies demonstrate that mercury can disrupt redox homeostasis and promote inflammatory signaling at the cellular level, extrapolation of these findings to clinically significant outcomes in individuals with amalgam fillings requires careful interpretation.

In recent years, increasing attention has been directed toward the evaluation of saliva as a non-invasive diagnostic fluid capable of reflecting both local and systemic pathological processes. Salivary biomarkers, including enzymes, acute-phase proteins, cytokines, and oxidative stress markers, provide valuable insight into inflammatory and toxicological processes occurring in the oral environment [12,13]. Among these, ceruloplasmin, a copper-binding acute-phase protein with antioxidant properties, has been associated with inflammatory status and oxidative stress regulation [14]. Similarly, interleukin-8 (IL-8), a pro-inflammatory chemokine involved in neutrophil recruitment and activation, plays a crucial role in gingival inflammation and periodontal tissue responses [15]. Alterations in the salivary levels of these molecules have been reported in some observational studies involving individuals with amalgam restorations. However, the available human data are primarily cross-sectional and demonstrate associations rather than definitive causal relationships. The magnitude and clinical significance of such biomarker variations remain under investigation.

In parallel with biochemical changes, several histopathological alterations of the oral mucosa have been reported in association with metallic restorations. These include epithelial hyperplasia, chronic inflammatory infiltrates, vascular changes, and lichenoid epithelial reaction adjacent to amalgam surfaces [16,17].

From a public health perspective, concerns regarding mercury toxicity have led to significant regulatory changes. The Minamata Convention on Mercury, supported by the World Health Organization (WHO), advocates for a global phase-down and eventual phase-out of amalgam use, encouraging the adoption of alternative restorative materials with improved biocompatibility profiles [18]. While several countries, particularly in Scandinavia, have already implemented strict restrictions or complete bans on dental amalgam, its use remains prevalent in parts of Central and Eastern Europe, especially with publicly funded dental care systems [19]. This geographic disparity underscores the continued clinical relevance of understanding the biological effects of amalgam restorations.

Given the persistence of amalgam restorations in the population and the growing emphasis on personalized and preventive oral healthcare, a comprehensive evaluation of their potential impact on oral tissues and salivary biochemistry remains warranted. Therefore, the aim of this narrative review is to synthesize and critically appraise current evidence regarding the potential effects of dental amalgam fillings on oral mucosal integrity and salivary enzymatic composition, with particular emphasis on inflammatory mediators, oxidative stress markers, and histopathological changes in the gingival and oral mucosa.

## 2. Methodology and Search Strategy

**Figure 1:**
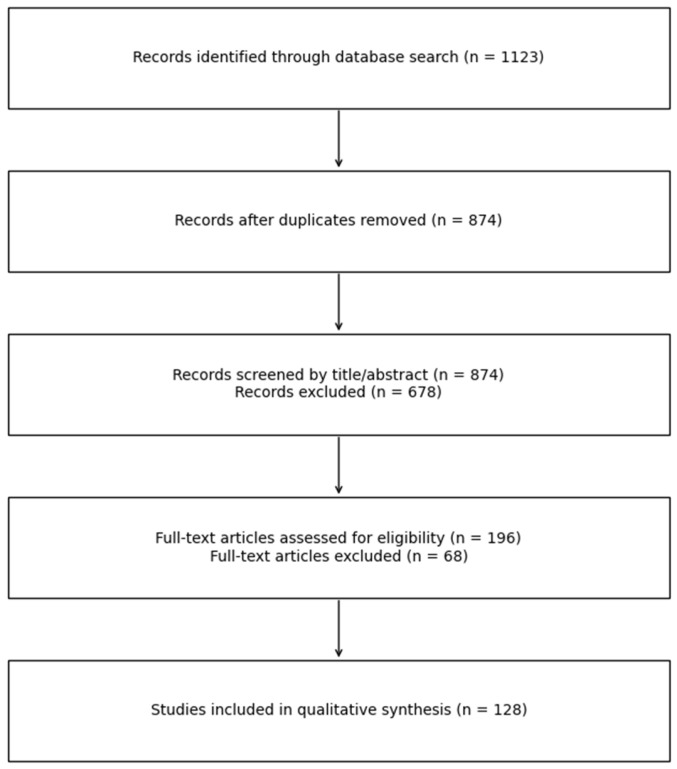
Simplified diagram summarizing the identification, screening, eligibility assessment, and inclusion of studies considered in this narrative review.

This manuscript represents a narrative review aimed at synthesizing current evidence regarding the biological, histopathological, clinical, and public health implications of dental amalgam exposure. Although not designed as a formal systematic review or meta-analysis, a structured and reproducible search strategy was employed to enhance methodological rigor and transparency. A comprehensive literature search was conducted in the PubMed/MEDLINE, Scopus, and Web of Science databases, covering publications from January 1990 to January 2026. Earlier landmark publications were additionally consulted to contextualize the historical development and long-term clinical use of dental amalgam. The search strategy combined relevant keywords and controlled vocabulary terms related to dental amalgam and its biological effects, including “dental amalgam,” “mercury release,” “oral mucosa,” “gingival inflammation,” “salivary biomarkers,” “oxidative stress,” “ceruloplasmin,” “interleukin-8,” “lichenoid lesions,” “histopathology,” and “mercury toxicity”. The initial search yielded 1123 records. After the removal of duplicate entries across databases, 874 unique titles remained. Titles and abstracts were screened for relevance to the topic of dental amalgam–related biological and clinical outcomes. At this stage, studies clearly unrelated to dental materials, articles focusing exclusively on occupational or high-dose environmental mercury exposure, and publications not addressing oral or systemic biological effects were excluded. Following this screening process, 196 articles were retained for full-text assessment. Full-text evaluation was performed to determine eligibility based on relevance to the objectives of the present review. Eligible publications included in vitro experimental studies investigating cellular and molecular mechanisms, animal studies assessing tissue or systemic responses, observational human studies (cross-sectional, case–control, and cohort designs), randomized clinical trials where available, and relevant systematic reviews. Non-English articles and conference abstracts without accessible full texts were excluded. After full-text assessment, 128 articles were considered directly relevant and were included in the qualitative synthesis. From these, studies providing the most methodologically robust, clinically relevant, or representative findings were selectively cited in the reference list (Figure 1).

Given the heterogeneity of study designs, populations, biomarker assessment methods, and outcome definitions, quantitative synthesis was not feasible and a formal meta-analysis was not undertaken. Instead, the evidence was analysed qualitatively, with explicit attention to study design, sample size, methodological limitations, and potential sources of bias. Particular emphasis was placed on distinguishing mechanistic evidence derived from in vitro and animal models from associative findings reported in human observational studies. Where available, longitudinal and randomized clinical data were prioritized in the interpretation of clinical relevance. Throughout the review, biological plausibility was carefully differentiated from demonstrated clinical causation. Potential confounding variables, including periodontal disease status, smoking, systemic inflammatory conditions, dietary habits, and alternative sources of mercury exposure, were considered when interpreting findings related to salivary biomarkers, histopathological changes, and epidemiological outcomes. This approach was intended to provide a balanced and scientifically grounded synthesis while acknowledging the methodological limitations and variability inherent in the existing body of literature.

## 3. Composition and Biological Properties of Dental Amalgam

Dental amalgam has been used for more than a century as a restorative material due to its durability, mechanical strength, and relatively low cost [1]. Historically, mixtures of mercury and metal alloys were described in early Chinese and later European medical texts, with widespread clinical adoption occurring in the nineteenth century despite initial controversy, including the so-called “Amalgam War” in the United States [1,20,21]. Over time, refinements in alloy composition improved its physical performance and corrosion resistance, contributing to its long-standing clinical success.

Dental amalgam is formed by combining elemental mercury with a powdered alloy primarily composed of silver, tin, and copper, with minor additions such as zinc and trace metals [22,23]. Mercury acts as a binding phase, allowing amalgamation of alloy particles and formation of a solid restorative material. After setting, the material consists of intermetallic phases resulting from the reaction between mercury and alloy components. In earlier low-copper formulations, the presence of the γ2 (Sn7Hg) phase was associated with increased corrosion susceptibility. The introduction of high-copper alloys significantly reduced or eliminated this phase through preferential tin–copper reactions, improving mechanical strength and marginal integrity [24,25].

From a biological perspective, the relevance of amalgam composition lies in its potential for corrosion and ion release under intraoral conditions. The oral cavity constitutes a dynamic environment characterized by fluctuations in pH, temperature, mechanical stress, and salivary composition. These factors may influence electrochemical reactions at the amalgam surface, leading to the gradual release of mercury vapor and metallic ions [26,27]. Although the material is generally considered clinically stable, it is not biologically inert. The presence of mercury, even in low concentrations, has prompted ongoing evaluation of its local and systemic interactions [4,5,6,27].

Importantly, regulatory classification of dental amalgam reflects recognition of both its clinical utility and the need for controlled handling and environmental management [28]. Thus, while the material’s composition underpins its mechanical advantages, it also forms the basis for continued investigation into its biological behavior within the oral environment.

## 4. Mechanisms of Mercury Release and Interaction with Oral Mucosa

Although dental amalgam is generally considered a stable restorative material, it is not biologically inert. Once placed in the oral cavity, amalgam restorations are continuously exposed to a complex environment characterized by mechanical forces, chemical fluctuations, enzymatic activity, and microbial metabolism. These factors contribute to the gradual release of mercury and other metallic components from the amalgam surface over time [27,29]. Understanding the mechanisms underlying this release is essential for interpreting the biological responses observed in oral tissues.

### 4.1. Mercury Release from Dental Amalgam

Mercury release from amalgam occurs primarily in two forms: elemental mercury vapor and mercury ions. Elemental mercury vapor is generated mainly through surface evaporation and is enhanced by physical stimuli such as mastication, bruxism, and thermal changes caused by hot food and beverages. Numerous experimental studies have demonstrated that chewing can transiently increase intraoral mercury vapor concentrations, which may subsequently be inhaled and absorbed through the respiratory epithelium or oral mucosa [30,31].

In parallel, electrochemical corrosion plays a key role in mercury ion release. The oral cavity functions as an electrolytic environment, with saliva acting as a conductive medium containing ions, proteins, and enzymes. Differences in electrochemical potential between amalgam phases or between amalgam and adjacent metallic restorations may promote galvanic reactions, accelerating corrosion processes. The breakdown of intermetallic phases leads to the liberation of mercury, silver, tin, and copper ions into saliva and surrounding tissues. pH fluctuations represent another important determinant of mercury release. Acidic conditions, commonly associated with cariogenic diets, gastroesophageal reflux, or plaque accumulation, have been shown to increase amalgam corrosion rates [32]. Additionally, salivary flow rate and buffering capacity influence the persistence and clearance of released ions, further modulating local exposure levels. Although corrosion processes and mercury vapor release are well documented under experimental conditions, the magnitude of exposure in vivo is generally low and influenced by multiple clinical factors, including restoration age, surface integrity, and individual oral environment. Therefore, the biological implications of released mercury should be interpreted in relation to actual exposure levels observed in clinical settings [33].

### 4.2. Absorption and Distribution in Oral Tissues

Once released, mercury can interact directly with oral soft tissues. The oral mucosa, particularly non-keratinized squamous epithelium, exhibits relatively high permeability, facilitating the diffusion of mercury species. Elemental mercury vapor readily crosses cell membranes due to its lipophilic nature, whereas ionic mercury interacts with sulfhydryl groups of proteins and enzymes, leading to intracellular accumulation [34]. Gingival tissues adjacent to amalgam restorations represent a site of prolonged exposure. Mercury has been detected in gingival biopsies, dental plaque, and saliva of individuals with amalgam fillings, supporting the concept of local deposition [30,31,35]. Macrophages and other immune cells may internalize mercury particles or ions, contributing to chronic inflammatory responses and sustained tissue irritation [36]. Systemically, absorbed mercury is distributed to various organs, including the kidneys and central nervous system, where it undergoes oxidation to divalent mercury [37,38]. While systemic toxicity remains a topic of debate at typical exposure levels from dental amalgam, localized effects within the oral cavity are more consistently documented, particularly in susceptible individuals. Most evidence regarding oxidative stress induction and protein interaction derives from in vitro or animal models using controlled exposure concentrations. While these findings provide mechanistic insight, direct extrapolation to clinically relevant oral tissue damage in humans remains limited and requires cautious interpretation [30,31,35].

### 4.3. Cellular and Molecular Effects

At the cellular level, mercury interferes with fundamental biological processes. One of its primary mechanisms of toxicity involves binding to thiol (-SH) groups, which are abundant in enzymes, structural proteins, and antioxidant molecules such as glutathione [34,39]. This interaction disrupts enzymatic activity and compromises cellular redox balance. Mercury exposure has been associated with mitochondrial dysfunction, characterized by impaired electron transport chain activity and increased production of ROS. Excessive ROS generation overwhelms antioxidant defence mechanisms, leading to oxidative damage to lipids, proteins, and nucleic acids [40,41,42]. These processes are particularly relevant in oral epithelial cells, which exhibit high metabolic turnover. In addition to oxidative stress, mercury can modulate immune signalling pathways. Experimental studies have shown that mercury exposure influences cytokine expression, enhances pro-inflammatory mediator release, and may promote hypersensitivity reactions [43,44,45]. In the oral cavity, these mechanisms can provide a possible biological basis for the development of lichenoid lesions and chronic gingival inflammation adjacent to amalgam restorations [46].

## 5. Salivary Biomarkers and Oxidative Stress Responses Associated with Dental Amalgam

Saliva has emerged as a valuable diagnostic medium in oral and systemic health research due to its accessibility, non-invasive collection, and close interaction with oral tissues. It reflects a dynamic equilibrium between local inflammatory processes, systemic physiological states, and environmental exposures. In the context of dental amalgam, salivary analysis offers unique insights into biochemical and immunological changes associated with chronic exposure to metallic components [12,13,14,15].

### 5.1. Saliva as a Diagnostic Tool in Amalgam-Related Research

The composition of saliva includes enzymes, cytokines, acute-phase proteins, antioxidants, and metal ions, all of which may be influenced by the presence of dental restorations. Salivary biomarkers have been widely investigated in periodontal disease, oral cancer, and toxicological studies, supporting their relevance as indicators of tissue responses [12,13,14,15].

Compared to blood-based biomarkers, salivary markers provide a more direct assessment of local oral conditions. This is particularly important when evaluating the biological impact of amalgam, as many of its effects are localized to the oral environment rather than systemic in nature [13,15].

### 5.2. Ceruloplasmin and Antioxidant Defense

Ceruloplasmin is a copper-containing glycoprotein primarily synthesized in the liver and secreted into plasma, but it is also detectable in saliva. It functions as an acute-phase reactant and plays a critical role in oxidative stress regulation through its ferroxidase activity, which limits free iron-mediated radical formation [47]. Several studies have reported altered salivary ceruloplasmin levels in inflammatory oral conditions. In the context of dental amalgam, increased ceruloplasmin concentrations have been interpreted as a compensatory response to enhanced oxidative stress induced by mercury exposure. Mercury′s affinity for thiol groups and its capacity to disrupt antioxidant enzymes may necessitate upregulation of alternative protective mechanisms, including ceruloplasmin-mediated pathways [14].

### 5.3. Interleukin-8 (IL-8) and Inflammatory Signaling

Interleukin-8 (IL-8) is a pro-inflammatory chemokine involved in neutrophil chemotaxis and activation [48]. It plays a central role in gingival inflammation and is consistently elevated in periodontal disease [49]. IL-8 expression may also be modulated by exposure to metals and oxidative stress. Experimental evidence suggests that mercury can activate transcription factors such as NF-kB, leading to increased cytokine production. Elevated salivary IL-8 levels observed in individuals with amalgam restorations may therefore reflect local immune activation driven by metal-induced cellular stress. Importantly, IL-8 alterations have been correlated with clinical parameters of gingival inflammation, supporting its relevance as a biomarker of tissue response [50].

### 5.4. Oxidative Stress Markers in Saliva

Beyond ceruloplasmin and cytokines, a broad spectrum of oxidative stress markers has been investigated in relation to dental amalgam. These include malondialdehyde (MDA), superoxide dismutase (SOD), catalase and glutathione-related enzymes. MDA is a product of lipid peroxidation, commonly used as an indicator of oxidative membrane damage [51]. SOD and catalase are enzymatic antioxidants responsible for detoxifying superoxide radicals and hydrogen peroxide, respectively [52]. Glutathione-related enzymes play a central role in cellular detoxification and redox regulation. Alterations in the activity or concentration of these markers have been reported in saliva samples from individuals with long-standing amalgam restorations. Increased oxidative stress marker levels may suggest a shift toward a pro-oxidative state; however, whether this reflects clinically meaningful tissue injury or a transient adaptive response remains uncertain [52,53].

### 5.5. Clinical Relevance of Salivary Changes

The clinical significance of salivary biomarker alterations lies in their potential to serve as early indicators of tissue stress before overt mucosal pathology becomes apparent. Chronic low-grade oxidative stress and inflammation may predispose individuals to persistent gingival irritation, impaired wound healing, and mucosal hypersensitivity reactions. Importantly, salivary biomarker profiles may vary based on individual susceptibility, genetic background, oral hygiene status, and duration of amalgam exposure. This variability underscores the need for personalized assessment rather than generalized assumptions regarding amalgam safety. Although statistically significant differences in salivary biomarkers have been reported in some studies comparing individuals with and without amalgam restorations, the magnitude of these alterations is generally modest. In many cases, biomarker levels remain within physiological or reference ranges, raising questions regarding their direct clinical significance. Therefore, while changes in oxidative stress markers and inflammatory mediators may reflect localized biological responses, they do not necessarily translate into overt clinical pathology in otherwise healthy individuals. The distinction between biochemical variation and clinically meaningful disease must be carefully maintained [53].

Interpretation of salivary biomarker findings must also consider potential confounding variables. Periodontal disease, even in subclinical form, is independently associated with elevated inflammatory cytokines such as IL-8 and with increased oxidative stress markers [15,49]. Smoking is another well-established modulator of salivary antioxidant systems and inflammatory mediators. Systemic inflammatory conditions, dietary factors, medication use, and psychosocial stress may further influence salivary composition. In addition, mercury exposure from non-dental sources, including dietary intake and environmental exposure, may contribute to overall mercury burden [49]. Many available studies do not comprehensively control for these variables, which limits the strength of causal inference regarding amalgam-specific effects. An additional methodological consideration relates to variability in saliva sampling procedures across studies. Differences between whole saliva and gland-specific saliva (parotid or submandibular/sublingual), as well as between stimulated and unstimulated collection protocols, may influence biomarker concentrations. Circadian variation, flow rate, and storage conditions can further affect the stability of oxidative stress markers and cytokines. Many studies do not uniformly report these parameters, which may contribute to heterogeneity in reported results and complicate direct comparison between investigations.

## 6. Histopathological Changes of the Gingiva and Oral Mucosa Associated with Dental Amalgam

The oral mucosa represents the first biological barrier exposed to restorative materials placed within the oral cavity. Due to its continuous renewal, vascularization, and intimate contact with saliva and dental surfaces, the oral epithelium is particularly sensitive to chronic chemical and mechanical stimuli. In the case of dental amalgam, long-term exposure to metallic ions and corrosion products has been associated with a range of histopathological alterations affecting both the epithelial and connective tissue compartments of the gingival and oral mucosa [16,17,54].

### 6.1. Epithelial Alterations

**Figure 2:**
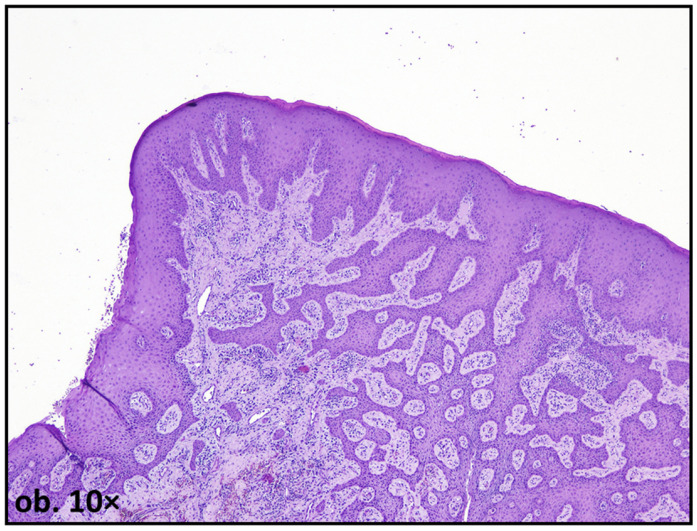
Oral mucosa adjacent to amalgam filling, showing extensive epithelial hyperplasia, with pseudoepitheliomatous pattern.

One of the most frequently reported histological findings in mucosal tissues adjacent to amalgam restorations is epithelial hyperplasia, sometimes with pseudoepitheliomatous appearance, characterized by increased thickness of the stratified squamous epithelium. This alteration is commonly interpreted as an adaptive response to persistent irritation, reflecting increased cellular proliferation aimed at reinforcing the epithelial barrier. In some cases, acanthosis and elongation of epithelial rete ridges have been observed, particularly in areas subjected to prolonged contact with amalgam surfaces [16,17] (Figure 2).

**Figure 3:**
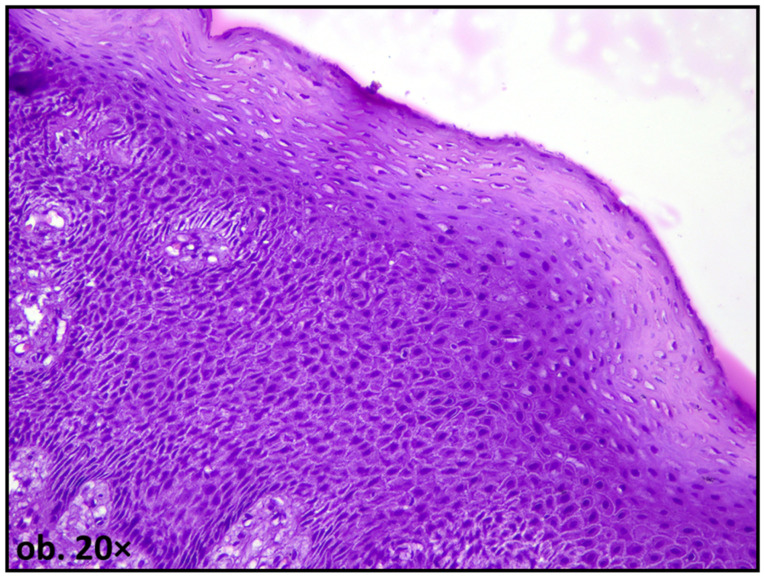
Oral mucosa adjacent to amalgam filling, showing spongiosis and reactive nuclear atypia.

In addition to hyperplastic changes, degenerative epithelial alterations have also been described. These may include intracellular edema (hydropic degeneration), intraepithelial edema (spongiosis), vacuolization of basal keratinocytes, and focal disruption of epithelial architecture, with reactive nuclear atypia [16] (Figure 3).

**Figure 4:**
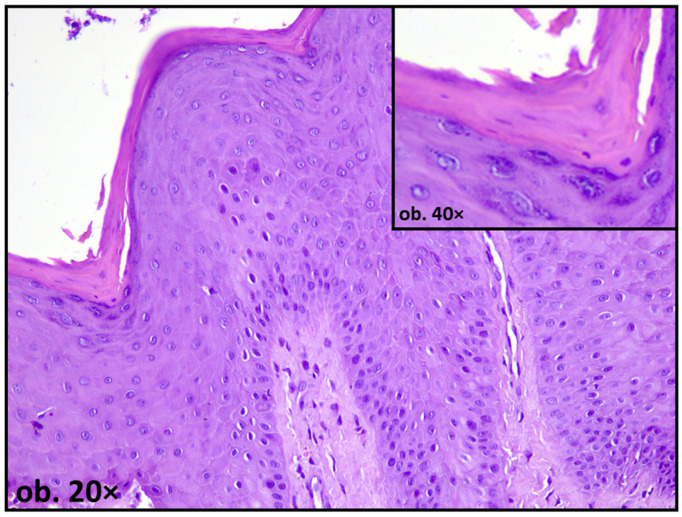
Oral mucosa adjacent to amalgam filling showing reactive changes—focal nuclear atypia and parakeratosis.

Such findings may be compatible with oxidative or metabolic stress; however, they are not specific and may also reflect nonspecific chronic irritation. Keratinization patterns may also be altered. While keratinization is generally protective, abnormal or excessive keratin deposition can indicate chronic irritation. In non-keratinized mucosa, focal parakeratosis has been reported in proximity to metallic restorations, suggesting a shift in epithelial differentiation pathways under sustained environmental stress [16,55] (Figure 4).

### 6.2. Connective Tissue and Inflammatory Changes

**Figure 5:**
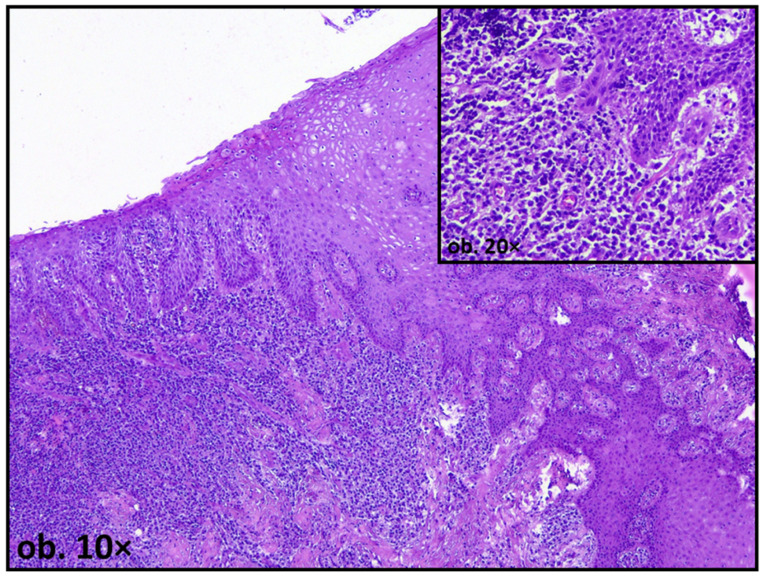
Oral mucosa adjacent to amalgam filling showing extensive chronic inflammatory infiltrate in the lamina propria, predominantly composed by lymphocites and plasma cells.

The lamina propria underlying the oral epithelium often exhibits chronic inflammatory infiltrate in tissues adjacent to amalgam restorations. Histologically, this infiltrate is typically composed of lymphocytes and plasma cells, with occasional macrophages and neutrophils, indicating a persistent, low-grade immune response rather than acute inflammation [56] (Figure 5).

**Figure 6:**
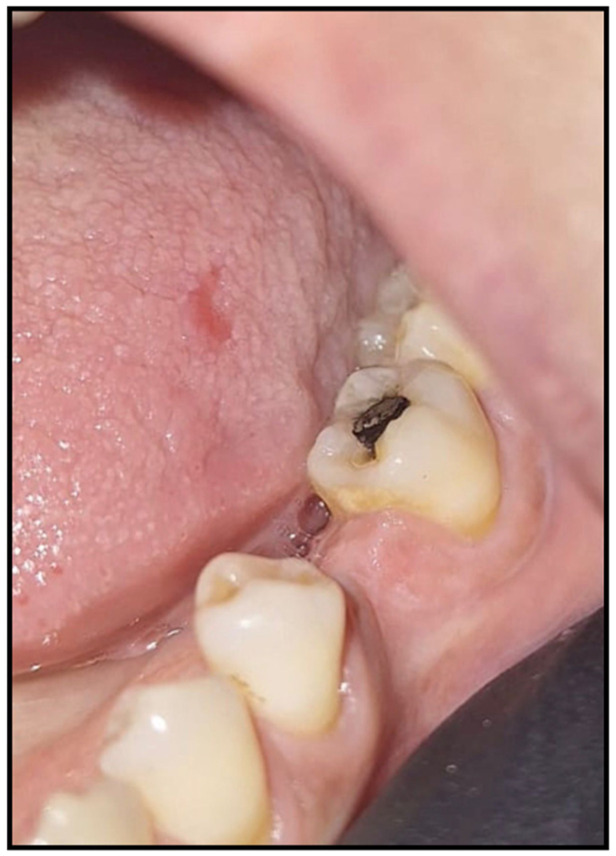
Mild gingival edema and tongue mucosal erosion, adjacent to amalgam filling.

Macrophages play a particularly important role in amalgam-associated tissue reactions. These cells may internalize metallic particles or ions released through corrosion processes, leading to prolonged antigen presentation and cytokine release. In some histological specimens, pigmented macrophages have been identified, suggesting phagocytosis of metal-derived deposits [54]. Vascular changes are another recurring feature. Dilated capillaries, endothelial activation, and increased vascular permeability have been documented, reflecting inflammatory signalling and tissue remodelling. Such changes may contribute to clinically observed erythema and edema in the gingival tissues surrounding amalgam restorations [46] (Figure 6).

### 6.3. Oral Mucosa Lichenoid Changes

**Figure 7:**
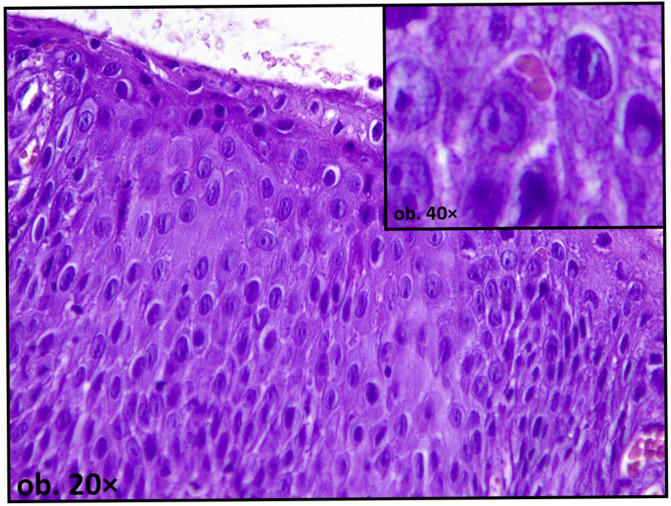
Epithelial reactive nuclear changes with Civatte bodies.

Among the most characteristic mucosal alterations associated with dental amalgam are oral lichenoid lesions. These lesions often present histopathological features resembling oral lichen planus, including a band-like lymphocytic infiltrate at the epithelium-connective tissue interface, basal cell degeneration, and apoptotic keratinocytes (Civatte bodies) [57] (Figure 7).

However, unlike idiopathic lichen planus, amalgam-associated lichenoid lesions tend to exhibit a topographical association with metallic restorations, frequently resolving or significantly reducing after replacement of the offending material. Nevertheless, not all lichenoid lesions in proximity to amalgam restorations demonstrate regression after replacement, and overlap with idiopathic oral lichen planus may complicate interpretation in clinical practice. Histologically, lichenoid reactions associated with amalgam may display asymmetry, deeper inflammatory infiltration, and a mixed cellular composition, distinguishing them from classic autoimmune lichen planus [46,56]. These features underscore the importance of careful histopathological and clinical correlation when evaluating mucosal lesions adjacent to amalgam restorations.

### 6.4. Ultrastructural and Molecular Considerations

At the ultrastructural level, mercury exposure has been shown to induce mitochondrial swelling and nuclear membrane irregularities in epithelial cells [58]. Such changes are consistent with impaired metabolism and increased oxidative stress, reinforcing findings from salivary biomarker studies. From a molecular perspective, histopathological alterations are often accompanied by changes in the expression of inflammatory mediators, adhesion molecules, and oxidative stress-related enzymes. Increased expression of cytokines and chemokines within gingival tissues may perpetuate immune cell recruitment, creating a self-sustaining inflammatory microenvironment [59]. These molecular events provide a mechanistic link between the biochemical alterations detected in saliva and the structural changes observed histologically. Together, they support the concept that chronic exposure to amalgam-derived components may lead to subtle but persistent tissue remodelling rather than overt cytotoxicity.

### 6.5. Clinical Correlation and Diagnostic Implications

Histopathological changes associated with dental amalgam are often subclinical or nonspecific, emphasizing the need for careful interpretation within a broader diagnostic framework. In many cases, patients may be asymptomatic or present with mild discomfort, burning sensations, or localized gingival inflammation. Importantly, the reversibility of certain lesions following amalgam removal has been reported, suggesting that early identification of amalgam-associated tissue changes may have practical therapeutic implications [56]. Histological evaluation, combined with salivary biomarker analysis and clinical assessment, may therefore contribute to a more comprehensive understanding of individual tissue responses to dental materials. It is important to emphasize that many of the histopathological changes described in tissues adjacent to amalgam restorations, such as epithelial hyperplasia, chronic inflammatory infiltrate, and vascular dilation, are not pathognomonic and may occur in response to various forms of chronic mechanical or chemical irritation. Therefore, the mere presence of such findings does not establish a specific etiological relationship with amalgam. Accurate diagnosis requires careful clinicopathological correlation, including lesion topography, temporal association with restorations, and, when relevant, improvement following material replacement. In particular, differentiation between oral lichenoid contact reactions and idiopathic oral lichen planus demands strict application of established diagnostic criteria. Although amalgam-associated lichenoid lesions often demonstrate a spatial relationship with metallic restorations and may regress after their removal, histological features alone are insufficient to confirm causation. Consequently, interpretation of these findings must remain cautious and individualized.

## 7. Clinical and Epidemiological Evidence

The interpretation of available evidence requires careful differentiation between levels of scientific investigation. Much of the mechanistic understanding regarding mercury-induced oxidative stress, immune activation, and mitochondrial dysfunction derives from in vitro and animal studies, which provide biological plausibility but do not directly establish clinical causation in humans. In contrast, most human data regarding dental amalgam exposure originate from cross-sectional or observational studies, which are able to demonstrate associations but cannot confirm cause–effect relationships. Longitudinal cohort studies and randomized clinical trials specifically addressing biological outcomes related to amalgam exposure remain comparatively limited. Therefore, conclusions regarding clinical impact must be framed within the context of this hierarchy of evidence.

From a restorative standpoint, evidence regarding the longevity of dental amalgam derives primarily from long-term cohort studies, retrospective clinical evaluations, and randomized controlled trials comparing amalgam with composite resin restorations. Several longitudinal investigations with follow-up periods exceeding 10–15 years have reported high survival rates of posterior amalgam restorations, often demonstrating superior marginal integrity and resistance to secondary caries compared with earlier generations of composite materials. These findings are supported by systematic reviews synthesizing clinical outcome data across diverse populations [60,61,62]. These observations underpin its continued presence in many public dental care systems, particularly in regions where low-cost solutions are necessary.

Reported prevalence rates of amalgam-associated oral lichenoid lesions vary substantially across studies and populations. In general population-based samples, prevalence is typically low, often reported between approximately 1% and 5%. Higher frequencies have been observed in referral-based clinical settings, where patients present with symptomatic mucosal lesions or suspected contact hypersensitivity. These differences likely reflect variations in diagnostic criteria, referral patterns, and study design rather than true population-level incidence. Importantly, the overall burden of clinically significant amalgam-associated lesions appears limited in the general population [63,64].

Epidemiological analyses have also examined the relationship between amalgam exposure and systemic biomarkers of mercury burden. Urinary mercury excretion has been correlated with the number and surface area of amalgam restorations, providing evidence of cumulative exposure. However, population-based studies consistently demonstrated that, for the majority of healthy adults, urinary mercury levels remain below thresholds associated with clinically significant systemic toxicity. This distinction highlights the importance of considering individual susceptibility, renal function, and concurrent exposure sources when interpreting these data. Although urinary mercury levels correlate with the number and surface area of amalgam restorations, most population-based studies report concentrations below established safety thresholds in healthy individuals. It is important to distinguish measurable exposure from clinically significant toxicity. The presence of detectable mercury biomarkers does not, in itself, indicate adverse health outcomes, and current epidemiological evidence does not demonstrate consistent systemic pathology attributable solely to dental amalgam in the general population [65].

Several studies have investigated the interaction between amalgam restorations and periodontal or gingival health. Observational data suggest that in patients with good oral hygiene, the presence of amalgam alone does not significantly increase the risk of generalized gingivitis or periodontitis. Conversely, in cases of suboptimal hygiene, amalgam surfaces can contribute to localized plaque accumulation, corrosion, and low-grade inflammatory responses. These findings reinforce the concept that the biological impact of amalgam is multifactorial, depending not only on material properties but also on behavioural and host factors [66].

From a longitudinal perspective, prospective cohort studies evaluating the replacement of amalgam restorations with alternative materials provide valuable insights. Several reports document improvement or resolution of oral lichenoid lesions following removal of the amalgam, supporting the role of metal hypersensitivity or local irritation in lesion pathogenesis. Conversely, in asymptomatic individuals, removal of amalgam purely for prophylactic purposes has not consistently demonstrated clinical benefit, emphasizing the need for individualized decision-making guided by symptomatology, lesion characteristics, and patient preferences [67].

Geographical differences in amalgam use and associated outcomes have also been observed. In Scandinavian countries, where regulatory policies have led to a marked reduction or near-complete phase-out of amalgam, population studies report lower incidence of amalgam-associated mucosal lesions and decreased systemic mercury exposure compared with countries in Central and Eastern Europe, where amalgam remains utilized in public dental services. These disparities provide a natural experiment illustrating the combined influence of material availability, healthcare policies, and clinical practice on population-level outcomes [68].

The clinical and epidemiological evidence portrays dental amalgam as a highly effective restorative material. At the same time, a subset of individuals may experience localized mucosal alterations or changes in biomarkers indicative of oxidative stress and inflammation. Interpretation of these findings must account for the predominance of observational designs and the inherent limitations of such studies. Interpretation of epidemiological data must acknowledge several methodological limitations. Many available studies are cross-sectional in design, limiting causal inference. Sample sizes are frequently modest, and follow-up periods may be insufficient to detect long-term outcomes. Furthermore, control for confounding variables—such as socioeconomic status, access to dental care, oral hygiene practices, smoking, dietary habits, and coexisting systemic conditions—is often incomplete. Diagnostic criteria for mucosal lesions may also vary across studies, contributing to heterogeneity in reported prevalence. These factors underscore the need for cautious interpretation and highlight the importance of well-designed longitudinal research to clarify unresolved questions. Taken together, the available clinical and epidemiological data suggest that while localized biological responses may occur in susceptible individuals, current evidence does not support a consistent pattern of clinically significant adverse effects in the general population.

## 8. Regulatory and Public Health Perspectives

The global regulation of dental amalgam has been influenced not only by its clinical performance but also by environmental and precautionary public health considerations. While extensive research has examined potential biological effects of mercury released from amalgam restorations, current regulatory initiatives have been driven primarily by efforts to reduce environmental mercury pollution and cumulative global mercury burden rather than by definitive evidence of widespread clinical harm in the general population.

The Minamata Convention on Mercury, adopted by the United Nations in 2013, represents the most significant international framework addressing mercury reduction [18]. Within this context, the phase-down of dental amalgam reflects a precautionary approach that integrates environmental sustainability, occupational exposure control, and long-term public health strategy. The emphasis on alternative restorative materials is therefore aligned with broader ecological and environmental health objectives, even though dental amalgam continues to be considered clinically acceptable for many patients under appropriate conditions.

National regulatory policies vary substantially, reflecting differences in healthcare infrastructure, economic considerations, environmental priorities, and interpretation of available evidence. In Scandinavian countries, near-complete phase-out policies have been implemented largely on environmental grounds. In contrast, in several Central and Eastern European regions, amalgam remains in use within publicly funded systems due to its cost-effectiveness and durability. This geographic variability underscores the complex interplay between scientific evidence, environmental policy, and healthcare accessibility. In parallel, regulatory bodies in individual countries have implemented diverse policies reflecting local priorities, technological capacities, and population needs. Scandinavian countries have largely prohibited dental amalgam use in routine practice, driven by both environmental and precautionary health concerns [68]. In contrast, Central and Eastern European countries continue to use amalgam, primarily due to its cost-effectiveness, longevity, and accessibility in resource-limited settings. This geographic variability underscores the interplay between clinical evidence, environmental policy, and socio-economic factors in shaping dental practice.

## 9. Integrative Insights

The evidence synthesized in this review highlights a complex and heterogeneous landscape regarding dental amalgam and oral tissue responses. While mechanistic and experimental data provide biological plausibility for oxidative stress, immune modulation, and localized tissue alterations in the presence of mercury, translation of these findings to consistent clinical outcomes in human populations remains limited. Human studies predominantly rely on observational and cross-sectional designs, which demonstrate context-dependent associations rather than uniform causal effects. Reported alterations in salivary biomarkers and histopathological findings are generally modest in magnitude and frequently influenced by individual susceptibility, local inflammatory status, oral hygiene practices, and broader environmental exposures. Many of the described mucosal changes are nonspecific and may overlap with responses to other chronic irritative stimuli.

Consequently, the literature does not support a singular or deterministic biological pathway linking dental amalgam to clinically significant oral pathology. Instead, the available data suggest that biological responses, when present, are heterogeneous and likely modulated by host factors and exposure context. This perspective underscores the importance of individualized clinical assessment and cautious interpretation of biomarker or histological findings in patients with amalgam restorations.

## 10. Conclusions

Dental amalgam remains a durable and cost-effective restorative material with a long record of clinical use. Experimental studies demonstrate that mercury can influence oxidative balance and inflammatory pathways at the cellular level, providing biological plausibility for localized tissue responses. In clinical settings, subtle alterations in salivary biomarkers and mild histopathological changes adjacent to amalgam restorations have been reported, particularly in susceptible individuals. However, the available human evidence is largely observational, and current clinical and epidemiological data do not demonstrate uniform or consistently clinically significant harm in the general population attributable solely to dental amalgam restorations. Reported biomarker variations are generally modest, and mucosal lesions such as lichenoid reactions appear to affect a limited subset of patients. Regulatory efforts to reduce amalgam use are primarily driven by environmental and precautionary considerations rather than definitive evidence of widespread clinical toxicity. In contemporary practice, decisions regarding amalgam placement or replacement should therefore be individualized and guided by clinical findings, patient-specific factors, and broader public health priorities. Further longitudinal studies with rigorous methodological control are needed to clarify long-term biological effects and better define risk in potentially susceptible populations. Overall, current evidence supports a balanced and evidence-based approach to the role of dental amalgam in modern dentistry.

## Data Availability

No new data were created or analyzed in this study.
